# Análise da Mortalidade por Múltiplas Causas na Insuficiência Cardíaca de Acordo com a Fração de Ejeção

**DOI:** 10.36660/abc.20240475

**Published:** 2025-09-11

**Authors:** Giovanni Possamai Dutra, Bruno Ferraz de Oliveira Gomes, Thiago Moreira Bastos da Silva, Leticia Souza Peres, Marco Antônio Netto Armando Rangel, João Luiz Fernandes Petriz, Plinio Resende do Carmo, Emilia Matos Nascimento, Basilio de Bragança Pereira, Gláucia Maria Moraes de Oliveira

**Affiliations:** 1 Universidade Federal do Rio de Janeiro Rio de Janeiro RJ Brasil Universidade Federal do Rio de Janeiro, Rio de Janeiro, RJ – Brasil; 2 Hospital Barra D’Or Rio de Janeiro RJ Brasil Hospital Barra D’Or, Rio de Janeiro, RJ – Brasil; 3 Universidade do Estado do Rio de Janeiro Rio de Janeiro RJ Brasil Universidade do Estado do Rio de Janeiro (UERJ), Rio de Janeiro, RJ – Brasil

**Keywords:** Insuficiência Cardíaca, Mortalidade, Causas de Morte

## Abstract

**Fundamento:**

A mortalidade por insuficiência cardíaca (IC) pode ser subestimada quando considerada apenas a causa básica nas declarações de óbito (DO). A análise das causas múltiplas de óbito permite uma avaliação mais abrangente dos fatores associados à mortalidade.

**Objetivo:**

Analisar as causas múltiplas de óbito hospitalar e tardio em pacientes com IC descompensada, de acordo com a fração de ejeção (FE) — reduzida (ICFEr), levemente reduzida (ICFElr) ou preservada (ICFEp).

**Métodos:**

Estudo retrospectivo de uma coorte prospectiva composta por pacientes internados por IC descompensada em uma unidade cardiointensiva de um hospital privado. Foram avaliadas as causas múltiplas de óbito, tanto hospitalar quanto tardio. Adotou-se nível de significância de 5% para a análise estatística.

**Resultados:**

Foram incluídos 519 pacientes, com média de idade de 74,87 ± 13,56 anos, sendo 57,6% do sexo masculino. As frequências de ICFEp, ICFElr e ICFEr foram de 25,4%, 27% e 47,6%, respectivamente. As doenças cardiovasculares foram as principais causas de óbito hospitalar e tardio nos três grupos, sem diferença estatística entre eles. As principais causas isoladas de morte, tanto hospitalar quanto tardia, foram septicemia (A41), IC (I50, I50.0, I50.9) e pneumonia (J12–J18). Na mortalidade tardia, observou-se diferença estatisticamente significativa para septicemia e pneumonia. As doenças respiratórias crônicas foram mais prevalentes entre os pacientes com FE mais reduzida (ICFEr e ICFElr). A análise de correspondência revelou associação entre causas circulatórias e ICFEr, causas neoplásicas e ICFEp, e causas endócrino-metabólicas e ICFElr.

**Conclusão:**

A análise das causas múltiplas de óbito demonstra elevada incidência de mortes não circulatórias em pacientes com IC descompensada, independentemente da FE, relacionadas sobretudo à idade avançada e à presença de comorbidades crônicas.

**Figure:**
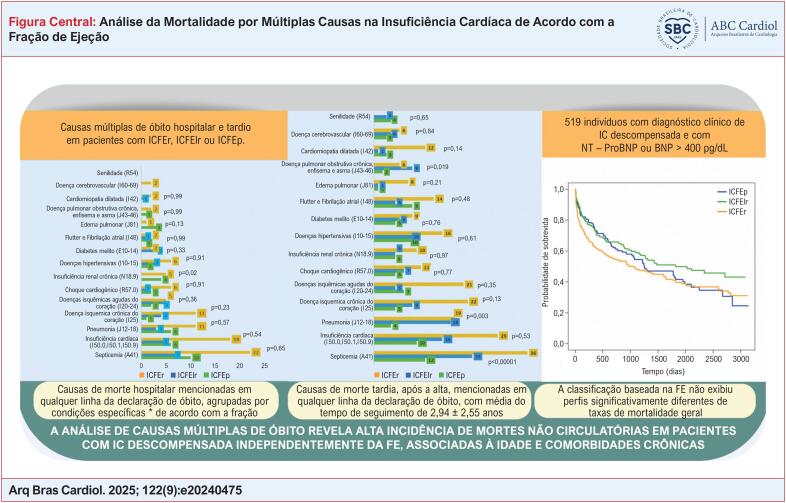
IC: insuficiência cardíaca; ICFEr: insuficiência cardíaca com fração de ejeção reduzida; ICFElr: insuficiência cardíaca com fração de ejeção levemente reduzida; ICFEp: insuficiência cardíaca com fração de ejeção preservada; FE: fração de ejeção; CID-10: Classificação Internacional de Doenças, 10ª Revisão. *Agrupamento conforme os códigos alfanuméricos da CID-10.

## Introdução

A insuficiência cardíaca (IC) é uma síndrome clínica complexa e de natureza sistêmica, caracterizada pela disfunção do coração em fornecer fluxo sanguíneo adequado para suprir as demandas metabólicas dos tecidos.^1^ Trata-se da terceira principal causa de morte por doenças cardiovasculares em países desenvolvidos e de uma relevante causa de morbidade e hospitalizações em todo o mundo.^2^ Atualmente, a IC apresenta uma prevalência global de 64,34 milhões de casos, correspondendo a 8,52 por 1.000 habitantes, com expressivo impacto em anos de vida saudável perdidos por incapacidade ( *years of healthy life lost due to disability* , YLDs). Nos últimos anos, a prevalência de IC aumentou 3,9% entre indivíduos com mais de 60 anos, consolidando-se como uma comorbidade de grande relevância nessa faixa etária.^3^

Atualmente, a IC é classificada de acordo com a fração de ejeção (FE) em três categorias: i) IC com FE reduzida (ICFEr), quando a FE é <40%; ii) IC com FE levemente reduzida (ICFElr), com FE entre 40% e 49%; e iii) IC com FE preservada (ICFEp), definida por FE ≥50%. Na classificação anterior, a ICFElr era denominada IC com FE intermediária. Estudos demonstram que a ICFElr apresenta características clínicas, estruturais e funcionais intermediárias entre a ICFEr e a ICFEp, o que fundamenta sua atual categorização. Este modelo é, atualmente, o principal adotado pelas diretrizes para orientar as recomendações terapêuticas.^4^

A análise isolada da causa básica de morte pode subestimar de forma significativa a mortalidade associada a doenças crônicas, como a IC. A avaliação das causas múltiplas de óbito constitui uma abordagem mais abrangente, permitindo identificar e correlacionar os determinantes envolvidos nos óbitos relacionados a uma condição específica.^5^

O objetivo deste estudo foi analisar as causas múltiplas de óbito, tanto hospitalar quanto tardio, em pacientes com IC classificados nos diferentes grupos de FE.

## Métodos

### Desenho do estudo

Trata-se de uma análise retrospectiva de um banco de dados coletado prospectivamente. Foram incluídos pacientes admitidos na unidade cardiointensiva de um hospital privado, com 162 leitos, maiores de 18 anos, no período de setembro de 2011 a junho de 2019. O projeto foi aprovado pelo Comitê de Ética em Pesquisa do Instituto D’Or de Pesquisa e Ensino (IDOR) sob parecer nº 3.582.453, emitido em 18/09/2019 (CAAE: 18502319.3.0000.5249).

### Critérios de elegibilidade

Foram incluídos pacientes internados por IC descompensada, com diagnóstico estabelecido com base em critérios clínicos clássicos, como os critérios de Framingham e Boston, além de marcadores laboratoriais, como peptídeo natriurético tipo B (BNP) e fragmento N-terminal do pró-peptídeo natriurético tipo B (NT-proBNP). Foram excluídas 203 internações múltiplas, sendo considerado apenas o último evento para cada paciente.

### Classificação dos pacientes

Os pacientes foram agrupados conforme a classificação atual da IC, baseada na FE obtida na primeira ecocardiografia realizada durante a internação: ICFEr (FE <40%), ICFElr (FE 40%-49%), ICFEp (FE ≥50%).^6^

### Coleta de dados sobre óbito

A data de óbito por todas as causas foi obtida no site da Corregedoria Geral da Justiça do Estado do Rio de Janeiro.^7^ As informações sobre causas múltiplas de óbito foram extraídas das DO fornecidas pela Secretaria Estadual de Saúde do Estado do Rio de Janeiro, por meio do Sistema de Informação sobre Mortalidade.

As causas declaradas de óbito foram agrupadas de acordo com a FE dos pacientes. Para a análise, todas as causas registradas nas DO foram consideradas, independentemente da linha ou da ordem de ocorrência. As causas foram organizadas conforme os capítulos dos códigos alfanuméricos da Classificação Internacional de Doenças, 10ª revisão (CID-10), visando a elaboração das tabelas de contingência e dos gráficos descritivos.

### Análise estatística

A normalidade das variáveis contínuas foi avaliada pelo teste de Kolmogorov-Smirnov. As variáveis contínuas foram descritas como média ± desvio padrão, e as categóricas, como número absoluto e percentual. A curva de Kaplan-Meier^8^ foi utilizada para estimar a sobrevida ao longo do tempo, com comparações entre os grupos realizadas pelo teste de Tarone-Ware.^9^ As associações entre as variáveis foram analisadas por meio do teste qui-quadrado de Pearson, aplicado nas tabelas e figuras.

Adicionalmente, realizou-se uma análise de correspondência para avaliar a relação entre os grupos de FE e os grupos de patologias classificados segundo a CID-10. Os gráficos fatoriais dessa análise foram construídos a partir das tabelas de contingência, que expressam a frequência observada das causas de óbito nos três grupos de IC. Essa abordagem permite representar, de forma gráfica, as associações entre os grupos de FE e as múltiplas causas de óbito, tanto hospitalar quanto tardio, destacando a interação entre as variáveis e os desfechos.

As análises foram conduzidas nos softwares IBM SPSS Statistics for Windows, versão 28.0^10^ e R.^11^ O nível de significância foi fixado em 5%.

## Resultados

Foram incluídos 519 pacientes, com média de idade de 74,87 ± 13,56 anos, dos quais 57,6% eram do sexo masculino. As distribuições dos pacientes nos grupos foram de 25,4% para ICFEp, 27% para ICFElr e 47,6% para ICFEr. O tempo médio de seguimento foi de 2,94 ± 2,55 anos.

Durante o período de acompanhamento, 52,3% dos pacientes evoluíram para óbito, sendo 14,5% durante a hospitalização. Não foram observadas diferenças estatisticamente significativas entre os grupos de FE quanto à mortalidade hospitalar.

A Tabela 1 apresenta as frequências absolutas e relativas das causas de óbito, agrupadas por sistemas orgânicos, conforme os capítulos da CID-10 e estratificadas pelos grupos de FE. As causas relacionadas ao aparelho circulatório (I) foram as mais prevalentes, especialmente no grupo ICFEr, seguidas pelas doenças do aparelho respiratório (J) e pelas causas infecciosas e mal definidas.

**Tabela 1:** Causas de morte mencionadas em qualquer linha da declaração de óbito, agrupadas por aparelho*, de acordo com a fração de ejeção de pacientes internados com diagnóstico de insuficiência cardíaca entre maio de 2011 e julho de 2019

Causas mencionadas por aparelhos ( * )	ICFEp (n, %)	ICFElr (n, %)	ICFEr (n, %)	Total de menções no DO (%)	Valor p
Aparelho circulatório (I)	77 (33,7%)	81 (34,3%)	193 (37,6%)	351 (35,9%)	0,50
Aparelho respiratório (J)	32 (14%)	38 (16,1%)	62 (12,1%)	132 (13,5%)	0,31
Infecciosas (A e B)	23 (10,1%)	33 (14%)	59 (11,5%)	115 (11,7%)	0,41
Mal definidas (R)	21 (9,2%)	21 (8,9%)	58 (11,3%)	100 (10,2%)	0,50
Aparelho geniturinário (N)	24 (10,5%)	17 (7,2%)	48 (9,4%)	89 (9,1%)	0,50
Neoplasias (C e D)	10 (4,4%)	10 (4,3%)	19 (3,7%)	39 (4,0%)	0,95
Endócrinas, nutricionais e metabólicas (E)	9 (4%)	12 (5%)	15 (2,9%)	36 (3,7%)	0,34
Sistema nervoso e cabeça (F, G e H)	9 (4%)	6 (2,5%)	11 (2,1%)	26 (2,6%)	0,38
Causas externas (V, X, W e Y)	8 (3,5%)	5 (2,1%)	17 (3,3%)	30 (3,1%)	0,61
Sistema osteomuscular e cutâneo (L e M)	2 (0,9%)	3 (1,3%)	2 (0,4%)	7 (0,7%)	0,39
Outros grupamentos (K, P, O, S e T)	13 (5,7%)	10 (4,3%)	29 (5,7%)	52 (5,3%)	0,69
Total	228 (100%)	236 (100%)	513 (100%)	977 (100%)	—

* Agrupamento conforme os códigos alfanuméricos da CID-10. Foi utilizado o teste qui-quadrado de Pearson. FE: fração de ejeção; IC: insuficiência cardíaca; ICFEp: IC com FE preservada; ICFElr: IC com FE levemente reduzida; ICFEr: IC com FE reduzida.

Foram analisadas 266 DO, totalizando 977 menções de causas de óbito. Houve uma perda de 7% dos dados devido à ausência ou inconsistências nas DO.

**Figura 1:**
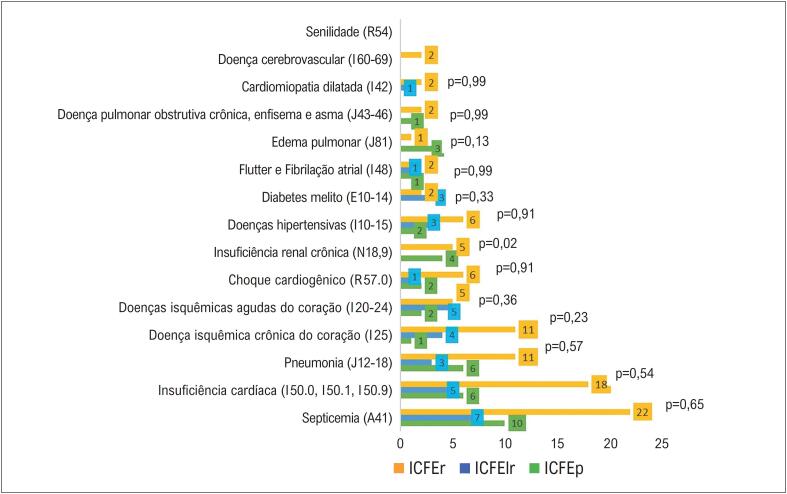
Causas de morte hospitalar mencionadas em qualquer linha da declaração de óbito, agrupadas por condições específicas * de acordo com a fração. Foi utilizado o teste qui-quadrado de Pearson. *Agrupamento por condições específicas, conforme os códigos alfanuméricos da CID-10. FE: fração de ejeção; IC: insuficiência cardíaca; ICFEp: IC com FE preservada; ICFElr: IC com FE levemente reduzida; ICFEr: IC com FE reduzida.

As causas de óbito hospitalar, mencionadas em qualquer linha da DO, estão apresentadas na Figura 1 , agrupadas por condições específicas, de acordo com a FE dos pacientes internados com diagnóstico de IC entre maio de 2011 e julho de 2019.

**Figura 2:**
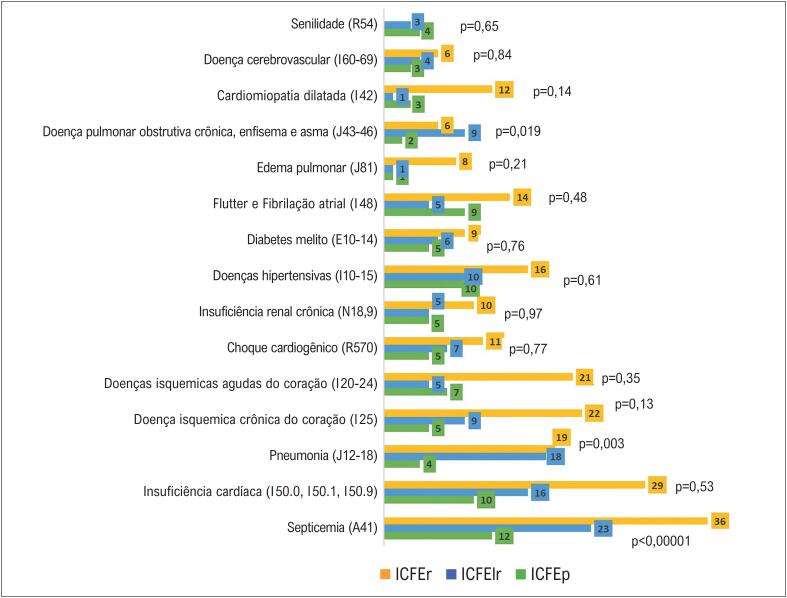
Causas de morte tardia, após a alta, mencionadas em qualquer linha da declaração de óbito, com média do tempo de seguimento de 2,94 ± 2,55 anos agrupadas por condições específicas,* de acordo com a fração de ejeção. Foi utilizado o teste qui-quadrado de Pearson. *Agrupamento por condições específicas, conforme os códigos alfanuméricos da CID-10. FE: fração de ejeção; IC: insuficiência cardíaca; ICFEp: IC com FE preservada; ICFElr: IC com FE levemente reduzida; ICFEr: IC com FE reduzida.

A Figura 2 apresenta as causas de óbito tardio, também mencionadas em qualquer linha da DO e agrupadas por condições específicas, segundo a FE dos pacientes incluídos no mesmo período.

**Figura 3:**
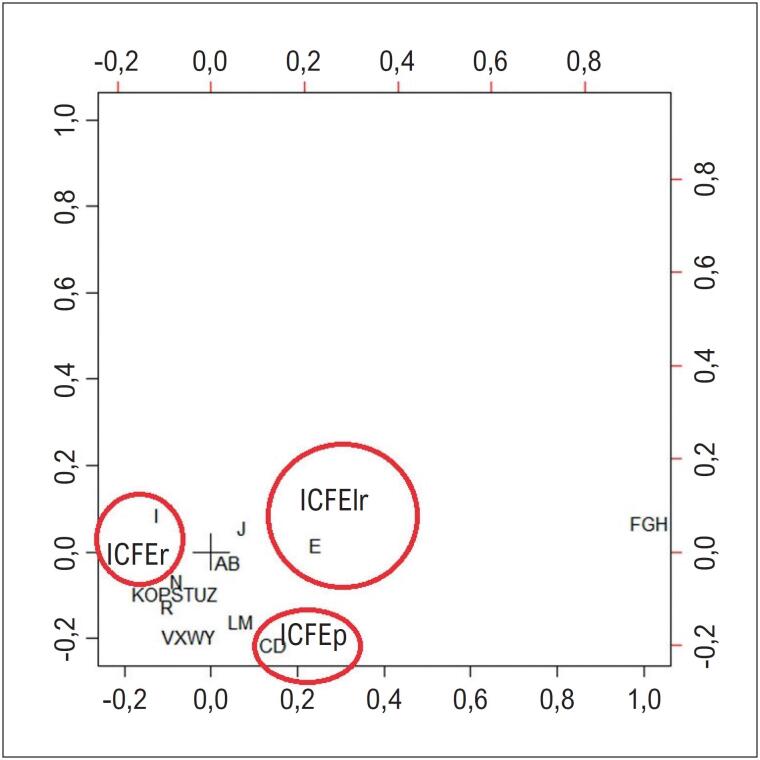
Análise de correspondência das causas de morte mencionadas em qualquer linha da declaração de óbito, agrupadas por aparelho, de acordo com a fração de ejeção de pacientes internados com diagnóstico de insuficiência cardíaca entre maio de 2011 e julho de 2019. FE: fração de ejeção; IC: insuficiência cardíaca; ICFEp: IC com FE preservada; ICFElr: IC com FE levemente reduzida; ICFEr: IC com FE reduzida.

A Figura 3 exibe a análise de correspondência das causas de morte, agrupadas conforme os capítulos da CID-10, da mesma forma que na Tabela 1 . Observa-se, na análise de correspondência, uma maior proximidade das causas relacionadas ao aparelho circulatório (I) com o grupo ICFEr, das causas neoplásicas (C e D) com ICFEp, e das doenças endócrino-metabólicas (E) com ICFElr.

**Figura 4:**
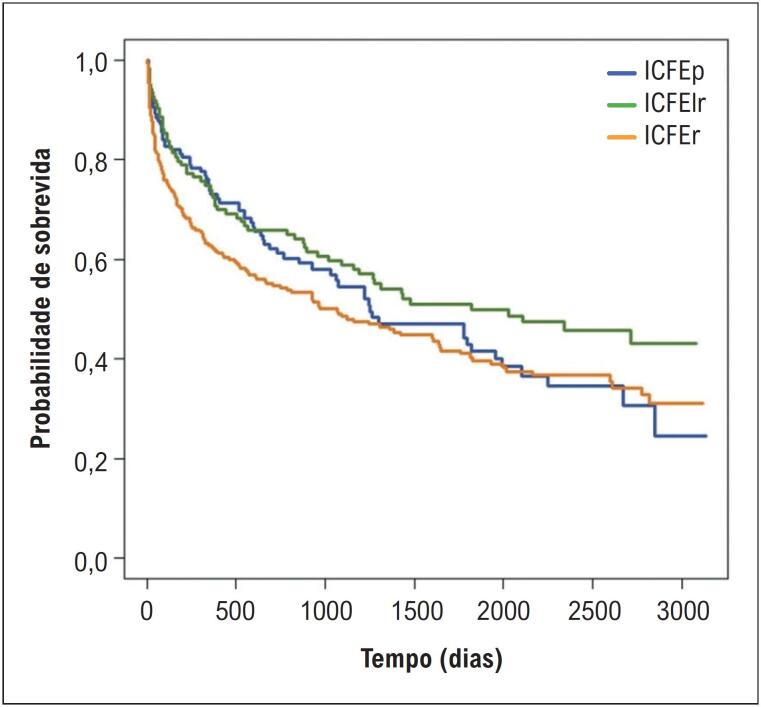
Curva de sobrevida de Kaplan-Meier^8^ dos pacientes com insuficiência cardíaca durante todo o período de seguimento. FE: fração de ejeção; IC: insuficiência cardíaca; ICFEp: IC com FE preservada; ICFElr: IC com FE levemente reduzida; ICFEr: IC com FE reduzida.

Na curva de sobrevida de Kaplan-Meier^7^ ( Figura 4 ), não foram identificadas diferenças estatisticamente significativas entre os grupos de IC.

## Discussão

A mortalidade em pacientes com IC é elevada e resulta de múltiplos fatores, como a FE, a presença de comorbidades e as causas específicas de óbito.^12 , 13^ No presente estudo, a classificação dos pacientes segundo a FE não demonstrou diferenças significativas nas taxas de mortalidade geral nem hospitalar em uma amostra com perfis clínicos variados. Por outro lado, observou-se que as principais causas de morte variaram entre os grupos de FE, o que reforça a heterogeneidade clínica e fisiopatológica dos pacientes com IC. Embora as causas circulatórias tenham sido as mais frequentes, elas corresponderam a apenas cerca de um terço dos óbitos no grupo ICFEr. Entre os pacientes com ICFElr, destacaram-se as doenças respiratórias crônicas como principais causas de morte. Além disso, a septicemia foi a causa específica mais prevalente, tanto na mortalidade hospitalar quanto na tardia, com maior incidência nos pacientes com FE mais baixa. Esses achados ressaltam a relevância da análise das causas múltiplas de óbito — uma abordagem pouco explorada na literatura —, capaz de revelar aspectos cruciais da trajetória clínica e do prognóstico dos pacientes com IC.^5^

A consideração isolada da causa básica de morte pode subestimar de forma relevante a mortalidade associada a doenças crônicas, como a IC. A análise das causas múltiplas de óbito, adotada neste estudo, representa uma abordagem mais abrangente para a avaliação da mortalidade, permitindo a identificação e a associação dos determinantes relacionados aos óbitos em função de uma doença específica.^5^ Dessa forma, este estudo contribui para o aprimoramento do conhecimento sobre a epidemiologia das causas de morte em pacientes com IC, além de fornecer subsídios para o desenvolvimento de estudos prospectivos que viabilizem a implementação de estratégias preventivas e terapêuticas mais adequadas aos diferentes perfis de FE. Corroborando esses achados, uma revisão recente demonstrou que a morte cardiovascular — especialmente a morte súbita e aquela relacionada diretamente à IC — foi responsável por mais da metade dos óbitos em pacientes com ICFEp, e por uma proporção ainda maior naqueles com FE abaixo da faixa normal. Contudo, as causas não cardiovasculares continuam representando um componente expressivo da mortalidade nessa população.^14^

A ICFEp é frequentemente associada a comorbidades crônicas não cardíacas, que impactam diretamente na fisiopatologia e no prognóstico da síndrome.^15^ Um achado relevante deste estudo foi a associação entre ICFEp e neoplasias, identificada na análise de correspondência. Esse resultado pode estar relacionado à elevada média de idade da amostra. A idade avançada, reconhecida como mais prevalente no grupo ICFEp em comparação ao ICFEr, tem sido consistentemente confirmada em estudos epidemiológicos.^16^ Dados recentes indicam que indivíduos que desenvolvem ICFEp possuem, em média, cerca de 6 anos a mais do que aqueles que desenvolvem ICFEr,^17^ além de a ICFEp representar uma proporção crescente dos casos incidentes de IC em pessoas idosas.

Entre os pacientes com IC, aproximadamente 10% a 25% apresentam ICFElr. Evidências recentes indicam que esses indivíduos apresentam um prognóstico intermediário entre ICFEr e ICFEp. A ICFElr está frequentemente associada a comorbidades crônicas não cardíacas, como hipertensão arterial, diabetes melito, doença renal crônica e anemia, que podem impactar negativamente os desfechos clínicos.^13^ Além disso, observou-se uma forte associação entre ICFElr e doenças respiratórias crônicas, como asma e doença pulmonar obstrutiva crônica, que foram significativamente mais prevalentes nesse grupo na presente amostra. A análise de correspondência também evidenciou uma relação entre ICFElr e doenças endócrino-metabólicas, especialmente o diabetes melito.

Pacientes com ICFEr apresentam elevado risco de morte por causas cardiovasculares, como infarto agudo do miocárdio, arritmias e choque cardiogênico.^13 , 18^ Nesta amostra, as causas cardiovasculares representaram aproximadamente 36% das menções nas DO, sendo mais frequentes no grupo ICFEr, com cerca de 20% das menções. Porém, causas não cardiovasculares também tiveram participação expressiva, destacando-se as doenças respiratórias (J) com 13,5%, as infecções (A e B) com 11,8% e as neoplasias (C e D) com 4%, totalizando aproximadamente 30% das causas mencionadas. Esse resultado difere dos dados da literatura nacional, que apontam que cerca de 50% dos óbitos em pacientes com IC estão relacionados ao aparelho circulatório (I) e 25% ao respiratório (J),^9^ o que pode justificar a elevada mortalidade observada nesta amostra. Os grupos ICFEp e ICFElr também apresentaram proporções relevantes de causas cardiovasculares, correspondendo a 7,9% e 8,3%, respectivamente. No entanto, ambos os grupos apresentaram participação expressiva de causas não cardiovasculares, como infecções (2,4% e 3,4%) e neoplasias (1,0% em ambos os grupos). No grupo ICFEr, manteve-se o predomínio de causas relacionadas ao aparelho circulatório, com 19,8% das menções. Esses achados estão em consonância com a literatura recente, que reconhece a septicemia como um fator de risco independente para mortalidade em pacientes com IC, especialmente naqueles com disfunção sistólica.^19^

A elevada frequência de causas infecciosas, tanto nos óbitos hospitalares quanto nos tardios, é um dado de grande relevância no contexto deste estudo, configurando-se, inclusive, como a causa mais prevalente de morte na amostra analisada. Corroborando esse achado, um estudo multicêntrico francês com 581 pacientes internados em unidades de terapia intensiva por IC aguda identificou uma taxa de infecção de 20% durante a hospitalização.^20^

A faixa etária a partir de 60 anos — próxima da idade mínima da amostra deste estudo —, associada à presença de processos infecciosos, contribui para o aumento da morbidade e da mortalidade desses pacientes quando comparados a indivíduos mais jovens.^20 , 21^ Em um estudo francês multicêntrico com 4.252 pacientes adultos, 429 desenvolveram infecção hospitalar. A ocorrência desse desfecho esteve independentemente associada à presença de comorbidades, neoplasias, neutropenia, uso prévio de antimicrobianos, internação em unidade de terapia intensiva, transferência de outro hospital, intubação traqueal por mais de 24 horas e tempo prolongado de hospitalização.^22^ Além disso, em pacientes idosos, a necessidade frequente de internação para manejo de suas condições clínicas é agravada pela alta letalidade das infecções adquiridas no ambiente hospitalar, o que torna esse fator particularmente relevante nessa população.^21^

Neste contexto, a idade avançada da amostra também influencia os padrões de ocorrência dos óbitos relacionados ao aparelho circulatório. Nos agrupamentos por condições específicas, observa-se que a IC (I50), que apresentou a maior proporção em um estudo brasileiro, com frequência de 23,26%,^5^ não foi a menção mais frequente nas DO desta amostra. No âmbito hospitalar, destacou-se uma maior proporção de menções a IC (I50) no grupo ICFElr em comparação ao grupo ICFEp. Além disso, observou-se um gradiente nas menções por IC (I50), por causas infecciosas e por doenças isquêmicas — tanto agudas quanto crônicas —, com variações nas frequências entre os grupos ICFEr, ICFElr e ICFEp, respectivamente. A presença recorrente dessas causas de morte reforça que o risco de mortalidade permanece elevado entre 12 e 18 meses após a hospitalização por IC.^23^ Adicionalmente, as taxas de readmissão por IC em adultos jovens são semelhantes às observadas em idosos, o que indica que o risco de reinternação persiste independentemente da faixa etária.^24^

A idade avançada também foi reconhecida como mais frequente no grupo ICFEp em comparação ao ICFEr, achado que tem sido consistentemente confirmado em diversos estudos epidemiológicos sobre ICFEp.^16^

Desde o século XX, observou-se um expressivo aumento da longevidade mundial, impulsionado principalmente pelo controle das doenças transmissíveis e pelo avanço das medidas sanitárias. Como consequência, houve um crescimento significativo na prevalência de doenças crônicas. No entanto, esse aumento da expectativa de vida é atenuado pelo envelhecimento e pelo crescimento populacional, resultando em elevação real das taxas de mortalidade por doenças cardiovasculares, associadas ao avanço da idade, ao maior consumo de tabaco e à adoção de dietas aterogênicas. Apesar desse cenário, observa-se, nas últimas décadas, uma redução das mortes por doenças cardiovasculares, atribuída ao avanço dos tratamentos específicos, o que tem contribuído para o prolongamento adicional da expectativa de vida.^25 , 26^ A IC é uma condição altamente prevalente, com incidência crescente, tendo como um dos principais fatores contribuintes o envelhecimento populacional. Dados epidemiológicos confirmam essa tendência e identificam a idade >65 anos como um fator predisponente para o desenvolvimento de IC.^27 - 33^

Em publicação anterior, utilizando a mesma amostra, observou-se que não houve diferença estatisticamente significativa na mortalidade entre os grupos de FE durante o seguimento de 2,94 ± 2,55 anos. Da mesma forma, a análise pela curva de sobrevida não identificou diferenças entre ICFEp e ICFElr, nem entre ICFEp e ICFEr, sendo observada apenas diferença significativa entre ICFElr e ICFEr ( Figura 4 ). Nesse estudo prévio, a hipótese de que a categorização da FE seria uma variável preditora de mortalidade hospitalar e tardia não foi confirmada, mesmo após a aplicação de modelos de *machine learning* . Ressalta-se que, na ocasião, as múltiplas causas de morte dessa população não foram analisadas, sendo objeto de investigação do presente estudo.^33^

As principais limitações deste estudo estão relacionadas à qualidade do preenchimento das DO, o que pode impactar a precisão das informações sobre as causas de morte. Outra limitação relevante foi a não localização de 21 DO da amostra, o que impossibilitou uma análise completamente abrangente. No entanto, foram obtidos dados de 266 DO (93% da amostra), totalizando 977 menções de causas de óbito. Esse quantitativo foi considerado suficiente e não comprometeu a interpretação clínica dos resultados.

Este estudo contribui para uma compreensão mais abrangente dos múltiplos fatores associados à IC, especialmente no que se refere à relação entre a FE e as doenças crônicas relacionadas ao envelhecimento. Até o momento, não foram identificados estudos que correlacionem mortalidade por múltiplas causas com os diferentes perfis de FE. A abordagem adotada permitiu aprofundar a compreensão dos mecanismos subjacentes à IC e às comorbidades associadas, além de gerar hipóteses que podem orientar o desenvolvimento de estratégias terapêuticas e de prevenção direcionadas à redução da morbidade e da mortalidade nessa população.

## Conclusão

Este estudo destaca a relevância da análise das causas múltiplas de óbito, capaz de revelar aspectos importantes da evolução clínica e do prognóstico de pacientes com IC, os quais não são captados pela análise isolada da causa básica de morte. Na amostra analisada, observou-se uma alta incidência de causas de morte — tanto hospitalares quanto tardias — não relacionadas ao aparelho circulatório, em pacientes internados por IC descompensada, independentemente da classificação em ICFEr, ICFElr ou ICFEp. Esses desfechos parecem estar mais associados à idade avançada e à presença de comorbidades crônicas do que propriamente à síndrome da IC. Esses achados reforçam a necessidade de estudos prospectivos para confirmar os resultados e para subsidiar o desenvolvimento de estratégias de manejo mais adequadas e personalizadas, considerando os diferentes fenótipos de IC.

Disponibilidade de Dados

Os conteúdos subjacentes ao texto da pesquisa estão contidos no manuscrito.

## *Material suplementar

Para informação adicional, por favor, clique aqui


