# Supplementary material for: Análise da Mortalidade por Múltiplas Causas na Insuficiência Cardíaca de Acordo com a Fração de Ejeção
Source: Arq Bras Cardiol. 2025 Sep 11;122(9):e20240475. [Article in Portuguese] doi: 10.36660/abc.20240475 (PMC12674839; doi:10.36660/abc.20240475)
Supplement: Supplementary file 1 [file 2024-0475_AO_material-suplementar.pdf]

Anexo 1

Causas de morte mencionadas em qualquer linha da declaração óbito, agrupadas por aparelho* de acordo com o gênero em pacientes internados com diagnóstico de IC entre maio de 2011 e julho de 2019.

**Causas mencionadas por aparelhos (*)**	Ocorrência de acordo com o gênero, número absoluto (%)		Total de menções na DO (%)
**Classificação da fração de ejeção**	Masculino	Feminino	
**Aparelho circulatório(I)**	196(20%)	155(15,9%)	351(35,9%)
**Aparelho respiratório (J)**	76(7,8%)	56(5,7%)	132(13,5%)
**Infecciosas (A e B)**	58(5,9%)	57(5,8%)	115(11,7%)
**Mal definidas (R)**	53(5,4%)	47(4,8%)	100(10,2%)
**Aparelho geniturinário(N)**	54(5,5%)	35(3,6%)	89(9,1%)
**Neoplasias (C e D)**	20(2,1%)	19(1,9%)	39(4,0%)
**Endócrinas Nutricionais e metabólicas (E)**	22(2,2%)	14(1,5%)	36(3,7%)
**Sistema Nervoso e cabeça (F, G e H)**	13(1,3%	13(1,3%)	26(2,6%)
**Causas externas (V, X, W e Y)**	16(1,7%)	14(1,4%)	30(3,1%)
**Sistema osteomuscular e cutâneo (L e M)**	4(0,4%)	3(0,3%)	7(0,7%)
**Outros Grupamentos (K, P, O, S e T)**	27(2,7%)	25(2,6%)	52(5,3%)
**Total**	539(55,2%)	438(44,8%)	977(100%)

Anexo 2

Causas de morte mencionadas em qualquer linha da declaração óbito, agrupadas por aparelho* de acordo com a fração de ejeção de pacientes internados com diagnóstico de IC entre maio de 2011 e julho de 2019.

Causas mencionadas por aparelhos (*)	Ocorrência de acordo com os tercis de idade, número absoluto (%)			Total de menções no DO (%)
Classificação da fração de ejeção Idade(anos)	1ºTercil (18 a 55)	2ºTercil (56 a 77)	3ºTercil (78 a 99)	
Aparelho circulatório(I)	24(2,4%)	112(11,5%)	215(22%)	351(35,9%)
Aparelho respiratório (J)	5(0,5%)	35(3,6%)	92(9,4%)	132(13,5%)
Infecciosas (A e B)	7(0,7%)	38(3,9%)	70(7,1%)	115(11,7%)
Mal definidas (R)	3(0,3%)	27(2,8%)	70(7,1%)	100(10,2%)
Aparelho geniturinário(N)	4(0,4%)	38(3,9%)	47(4,8%)	89(9,1%)
Neoplasias (C e D)	1(0,1%)	23(2,4%)	15(1,5%)	39(4,0%)
Endócrinas Nutricionais e metabólicas (E)	2(0,2%)	14(1,5%)	20(2%)	36(3,7%)
Sistema Nervoso e cabeça (F, G e H)	0(0%)	5(0,5%)	21(2,1%)	26(2,6%)
Causas externas (V, X, W e Y)	3(0,3%)	11(1,1%)	16(1,7%)	30(3,1%)
Sistema osteomuscular e cutâneo (L e M)	0(0%)	3(0,3%)	4(0,4%)	7(0,7%)
Outros Grupamentos (K, P, O, S e T)	6(0,6%)	22(2,2%)	24(2,5%)	52(5,3%)
Total	55(5,6%)	328(33.6%)	594(60,8%)	977(100%)
