# Supplementary material for: Análise da Mortalidade por Múltiplas Causas na Insuficiência Cardíaca de Acordo com a Fração de Ejeção
Source: Arq Bras Cardiol. 2025 Sep 11;122(9):e20240475. [Article in Portuguese] doi: 10.36660/abc.20240475 (PMC12674839; doi:10.36660/abc.20240475)
Supplement: Translation

# Translation: Analysis of Mortality from Multiple Causes in Heart Failure Categorized by Ejection Fraction

**DOI:** 10.36660/abc.20240475i

**Authors:** Giovanni Possamai Dutra, Bruno Ferraz de Oliveira Gomes, Thiago Moreira Bastos da Silva, Leticia Souza Peres, Marco Antônio Netto Armando Rangel, João Luiz Fernandes Petriz, Plinio Resende do Carmo, Emilia Matos Nascimento, Basilio de Bragança Pereira, Gláucia Maria Moraes de Oliveira

**Affiliations:** 1 Rio de Janeiro RJ Brazil Universidade Federal do Rio de Janeiro, Rio de Janeiro, RJ – Brazil; 2 Rio de Janeiro RJ Brazil Hospital Barra D’Or, Rio de Janeiro, RJ – Brazil; 3 Rio de Janeiro RJ Brazil Universidade do Estado do Rio de Janeiro (UERJ), Rio de Janeiro, RJ – Brazil

**Keywords:** Heart Failure, Mortality, Cause of Death

## Abstract

**Background:**

Mortality in heart failure (HF) may be underestimated when analyses rely solely on the underlying cause of death from death certificates (DCs), whereas including multiple causes of death enables a broader examination of mortality and its determinants.

**Objective:**

To analyze the multiple causes of in-hospital and late death in patients with decompensated HF and reduced ejection fraction (EF) (HFrEF), mildly reduced EF (HFmrEF), and preserved EF (HFpEF).

**Methods:**

Retrospective analysis of a prospective cohort of patients admitted for decompensated HF to a cardiac intensive care unit at a private hospital. The analysis included multiple causes of in-hospital and late deaths. A significance level of 5% was adopted.

**Results:**

The analysis included 519 individuals with a mean age of 74.87 ± 13.56 years, of whom 57.6% were male. The distribution of HFpEF, HFmrEF, and HFrEF was 25.4%, 27%, and 47.6%, respectively. Cardiovascular diseases (I) were the main causes of in-hospital and late death across all three EF groups, with no significant differences among them. The primary isolated causes of in-hospital and late death were septicemia (A41), HF (I50, I50.0, I50.9), and pneumonia (J12-J18). In late death, septicemia and pneumonia showed significant differences among the groups. Chronic respiratory causes were more frequent in patients with lower EF (HFrEF and HFmrEF). Correspondence analysis revealed an association between circulatory causes and HFrEF, neoplastic causes and HFpEF, and endocrine and metabolic causes and HFmrEF.

**Conclusion:**

The analysis of multiple causes of death reveals a high rate of non-circulatory deaths in patients with decompensated HF, regardless of EF, linked to age and chronic comorbidities.

## Introduction

Heart failure (HF) is a complex, systemic clinical syndrome defined as a cardiac dysfunction that causes an inadequate blood supply to meet the metabolic needs of tissues.^1^It is the third leading cause of death from cardiovascular disease in developed countries and an important cause of morbidity and hospitalization worldwide.^2^The global prevalence of HF currently stands at 64.34 million cases (8.52 per 1,000 inhabitants), significantly contributing to years of healthy life lost due to disability (YLDs). In recent years, the prevalence of HF has increased by 3.9% in patients older than 60 years of age, representing an important comorbidity in this age group.^3^

Currently, HF is classified based on EF into HF with reduced EF (HFrEF; EF < 40%), HF with mildly reduced EF (HFmrEF; EF between 40% and 49%), and HF with preserved EF (HFpEF; EF ≥ 50%). In a previous classification, HFmrEF was called HF with intermediate EF. Studies confirm that HFmrEF has characteristics that are intermediate between HFrEF and HFpEF, justifying the new categorization. This model has become the primary framework used by guidelines to provide treatment recommendations.^4^

Analyzing only the underlying cause of death can significantly underestimate mortality related to chronic diseases like HF. Including multiple causes of death provides a more comprehensive approach to evaluating mortality, allowing for a better correlation between the determinants associated with deaths caused by a specific condition.^5^

**Figure:** EF: ejection fraction; HF: heart failure; HFmrEF: HF with mildly reduced EF; HFpEF: HF with preserved EF; HFrEF: HF with reduced EF; ICD-10: International Classification of Diseases, 10^th^Revision. *Grouping according to ICD-10 alphanumeric codes.

The aim of this study was to analyze the multiple causes of in-hospital and late deaths in patients with HF classified by different EF groups.

## Methods

This was a retrospective analysis of a prospectively collected database. The study included patients admitted to the cardiac intensive care unit of a 162-bed private hospital, aged 18 years or older, between September 2011 and June 2019. The HF diagnosis on admission was based on classical clinical criteria, including the Framingham and Boston criteria, along with laboratory criteria such as BNP and NT-pro-BNP levels. The patients were grouped according to the current HF classification, *i.e.,* HFrEF, HFmrEF, and HFpEF, based on the first echocardiogram obtained during hospitalization.^6^In patients with more than one recorded event, only the most recent hospitalization was considered. Thus, 203 multiple hospitalizations were excluded, with only the last hospitalization being considered. The date of death from all causes was collected from the website of the General Internal Affairs Office of the Court of Justice of the State of Rio de Janeiro.^7^The data on mortality from multiple causes were collected from death certificates (DCs) provided by the State Health Department of Rio de Janeiro (SES-RJ) through the Mortality Information System (SIM). The occurrences of the reported causes of death were grouped according to EF. Multiple causes of death were considered—regardless of the line on which they were recorded on the DC or their order of occurrence—for constructing the contingency table and graphs. The causes were categorized based on the chapter groupings defined by the alphanumeric codes of the International Classification of Diseases, 10th Revision (ICD-10)

The project was submitted to the Research Ethics Committee of the *Instituto D’Or de Ensino e Pesquisa* (IDOR) and received approval based on the opinion issued on September 18, 2019, with ethics assessment number 18502319.3.0000.5249 (Opinion: 3.582.453).

### Statistical analysis

The normality of quantitative variables was assessed using the Kolmogorov-Smirnov test. The results were displayed as mean ± standard deviation (continuous variables) or number of occurrences and percentage (categorical variables). A Kaplan-Meier curve^8^was used to analyze survival over time, with the Tarone-Ware test^9^applied for group comparisons. Pearson's chi-square test was performed to verify the associations shown in the tables and figures. The analyses were performed using SPSS and R software.^10 , 11^A significance level of 5% was adopted.

A correspondence analysis was performed with the EF groups of HF and the pathology groups derived from the ICD-10. The factor plots from the correspondence analysis were constructed based on contingency tables, which show the observed frequency of causes of death across the three HF groups. The visual representation through correspondence factor analysis graphically displays the relationship between the variables of interest, such as EF classes, and their outcomes, including multiple causes of in-hospital and late death, illustrating how these variables relate to the outcomes.

## Results

The analysis included 519 individuals with a mean age of 74.87 ± 13.56 years, of whom 57.6% were male. The frequency distributions of the HFpEF, HFmrEF, and HFrEF groups were 25.4%, 27%, and 47.6%, respectively. The mean follow-up duration was 2.94 ± 2.55 years. During the follow-up period, 52.3% of the patients died, with 14.5% of them dying during hospitalization, without a significant difference among the groups.

Table 1 displays the absolute and relative frequencies of causes of death grouped by system and organized by EF. Causes related to the circulatory system (I) were the most frequent, especially in the HFrEF group, followed by those related to the respiratory system (J), and infectious and ill-defined causes. The data were collected from 266 death certificates, with a total of 977 entries. There was a 7% data loss from the DCs.

**Table 1:** Causes of death recorded in any line of the death certificate, grouped by system* and organized according to ejection fraction, among patients hospitalized with a diagnosis of HF between May 2011 and July 2019

Causes reported by systems ( * )	HFpEF (n, %)	HFmrEF (n, %)	HFrEF (n, %)	TOTAL (%)	p
Circulatory system (I)	77 (33.7%)	81 (34.3%)	193 (37.6%)	351 (35.9%)	0.50
Respiratory system (J)	32 (14%)	38 (16.1%)	62 (12.1%)	132 (13.5%)	0.31
Infectious (A and B)	23 (10.1%)	33 (14%)	59 (11.5%)	115 (11.7%)	0.41
Ill-defined (R)	21 (9.2%)	21 (8.9%)	58 (11.3%)	100 (10.2%)	0.50
Genitourinary system (N)	24 (10.5%)	17 (7.2%)	48 (9.4%)	89 (9.1%)	0.50
Neoplasms (C and D)	10 (4.4%)	10 (4.3%)	19 (3.7%)	39 (4.0%)	0.95
Endocrine, nutritional, and metabolic (E)	9 (4%)	12 (5%)	15 (2.9%)	36 (3.7%)	0.34
Nervous system and head (F, G, and H)	9 (4%)	6 (2.5%)	11 (2.1%)	26 (2.6%)	0.38
External causes (V, X, W, and Y)	8 (3.5%)	5 (2.1%)	17 (3.3%)	30 (3.1%)	0.61
Musculoskeletal and cutaneous system (L and M)	2 (0.9%)	3 (1.3%)	2 (0.4%)	7 (0.7%)	0.39
Other groups (K, P, O, S, and T)	13 (5.7%)	10 (4.3%)	29 (5.7%)	52 (5.3%)	0.69
Total	228 (100%)	236 (100%)	513 (100%)	977 (100%)	-

* Groups according to the ICD-10 alphanumeric codes. Pearson's chi-square test was applied. EF: ejection fraction; HF; heart failure; HFpEF: heart failure with preserved ejection fraction; HFmrEF: heart failure with mildly reduced ejection fraction; HFrEF: heart failure with reduced ejection fraction.

*Groups according to the ICD-10 alphanumeric codes. Pearson's chi-square test was applied. EF: ejection fraction; HF; heart failure; HFpEF: heart failure with preserved ejection fraction; HFmrEF: heart failure with mildly reduced ejection fraction; HFrEF: heart failure with reduced ejection fraction.

Figure 1 shows the causes of in-hospital deaths listed in any line of the DC, grouped by specific conditions and organized according to EF in patients admitted with a diagnosis of HF between May 2011 and July 2019.

**Figure 1:** Causes of in-hospital death mentioned on any line of the death certificate, grouped by specific conditions* according to the fraction. Pearson's chi-square test was used. *Grouping by specific conditions, according to ICD-10 alphanumeric codes. EF: ejection fraction; HF: heart failure; HFpEF: heart failure with preserved ejection fraction; HFmrEF: heart failure with mildly reduced ejection fraction; HFrEF: heart failure with reduced ejection fraction.

Figure 2 shows the causes of late deaths listed in any line of the DC, grouped by specific conditions and organized according to EF in patients admitted with a diagnosis of HF between May 2011 and July 2019.

**Figure 2:** Causes of late death after discharge, listed in any line of the death certificate, during a mean follow-up of 2.94 ± 2.55 years, grouped by specific conditions* and organized according to ejection fraction. Pearson's chi-square test was applied. EF: ejection fraction; HF: heart failure; HFpEF: heart failure with preserved ejection fraction; HFmrEF: heart failure with mildly reduced ejection fraction; HFrEF: heart failure with reduced ejection fraction.

Figure 3 illustrates the correspondence analysis of the causes of death grouped according to ICD-10 alphanumeric code by system, as in Table 1 . In the correspondence analysis, a proximity was observed between circulatory system causes (I) and HFrEF, between neoplastic causes (C and D) and HFpEF, and between endocrine and metabolic causes (E) and HFmrEF.

**Figure 3:** Correspondence analysis of the causes of death mentioned in any line of the death certificate, grouped by system (according to ICD-10 alphanumeric codes) and organized according to ejection fraction in patients hospitalized with a diagnosis of HF between May 2011 and July 2019. HFpEF: heart failure with preserved ejection fraction; HFmrEF: heart failure with mildly reduced ejection fraction; HFrEF: heart failure with reduced ejection fraction.

In the Kaplan-Meier survival curve^7^( Figure 4 ), no difference was observed among the HF groups.

**Figure 4:** Survival (Kaplan-Meier curve^8^) of patients with HF throughout the follow-up period. HFpEF: heart failure with preserved ejection fraction; HFmrEF: heart failure with mildly reduced ejection fraction; HFrEF: heart failure with reduced ejection fraction.

## Discussion

The mortality among patients with HF is high and depends on several factors, including EF, associated comorbidities, and causes of death.^12 , 13^The present study demonstrated that the classification based on EF did not exhibit significantly different patterns of overall or in-hospital mortality rates in a sample with diverse profiles. However, the main causes of death varied between EF groups, suggesting that there is clinical and pathophysiological heterogeneity among patients with HF. Despite being more frequent, circulatory causes accounted for only approximately one-third of deaths in patients with HFrEF. Chronic respiratory diseases were the most common causes of HFmrEF. Finally, septicemia was the most prevalent specific cause of both in-hospital and late mortality, with a higher frequency in patients with lower EF. Thus, the study highlighted the importance of analyzing multiple causes of death, a rare approach in the literature, as it can reveal relevant aspects of the clinical evolution and prognosis in patients with HF.^5^

Focusing solely on the underlying cause of death may substantially underestimate the mortality associated with chronic diseases such as HF. The assessment of multiple causes of death used in this study provides a more comprehensive approach to analyzing mortality, enabling the examination of the association between the determinants of death from a specific disease.^5^Thus, the study contributes to the knowledge of the epidemiology of causes of death in patients with HF and to the development of prospective studies that enable preventive and therapeutic strategies appropriate for each EF profile. In another recent review, cardiovascular death—particularly sudden death and death associated with HF—accounted for more than half of the deaths in patients with HFpEF and an even higher proportion in those with EF below the normal range. Non-cardiovascular death remains an important contributor to death.^14^

Notably, HFpEF is often accompanied by chronic non-cardiac comorbidities, which contribute to the pathophysiology and prognosis of the syndrome.^15^Another fact that should be highlighted in this study is the association of HFpEF with neoplasms in the correspondence analysis. One factor that may have contributed to the finding is the high average age of this sample. Older age has also been recognized as more frequent in HFpEF compared with HFrEF, which has been repeatedly confirmed in epidemiological studies of HFpEF.^16^Recent data suggest that individuals who develop HFpEF are, on average, 6 years older than those who develop HFrEF,^17^and HFpEF accounts for a greater proportion of incident HF cases in older individuals.

Approximately 10–25% of patients with HF have HFmrEF. According to the most recent evidence, these patients have an intermediate prognosis between HFrEF and HFpEF. Of note, HFmrEF is often associated with chronic non-cardiac diseases, such as hypertension, diabetes mellitus, chronic kidney disease, and anemia, which can affect the outcome of patients with HF.^13^Furthermore, HFmrEF is strongly associated with chronic respiratory diseases, such as asthma and chronic obstructive pulmonary disease (COPD), which were more prevalent in the HFmrEF group in this sample, with a statistically significant difference. The correspondence analysis also showed a relationship between HFmrEF and endocrine and metabolic diseases, such as diabetes mellitus.

Patients with HFrEF have a high risk of death from cardiovascular causes, such as acute myocardial infarction, cardiac arrhythmias, and cardiogenic shock.^13 , 18^In this sample, cardiovascular causes accounted for approximately 36% of the causes listed in the DCs and were more frequent in patients with HFrEF, with approximately 20% of the entries. However, other non-cardiovascular causes were also relevant, including respiratory diseases (J; 13.5% of the total), infections (A and B; 11.8%), and neoplasms (C and D; 4%), totaling around 30% of the listed causes. This finding differs from others in the national literature, in which approximately 50% of the deaths in patients with HF are attributed to the circulatory system (I) and 25% to the respiratory system (J),^9^probably explaining the increased mortality observed in this sample. Patients with HFpEF and HFmrEF also had an important proportion of cardiovascular causes, accounting for 7.9% and 8.3%, respectively. However, these groups also presented an important proportion of other non-cardiovascular causes, such as infections (A and B; 2.4% and 3.4%, respectively) and neoplasms (C and D; 1.0% each). Patients with HFrEF had a predominance of causes related to the circulatory system, corresponding to 19.8%. These findings are in line with the current literature, which points to septicemia as an independent risk factor for mortality in patients with HF, especially in those with systolic dysfunction.^19^

The high frequency of infectious causes in groups with specific conditions related to hospital admission and late deaths is also important in this context, as it was the most common cause of death in this sample. A French multicenter study of 581 patients admitted to intensive care units with acute HF revealed a 20% infection rate among them.^20^The age range of 60 years, which is close to the minimum age of the present sample, along with the infectious process, increased the morbidity and mortality of these patients compared to younger individuals.^20 , 21^In another multicenter French study with 4,252 adult patients, 429 developed in-hospital infections. The presence of comorbidities, neoplasia, neutropenia, previous use of antimicrobial agents, admission to an intensive care unit, transfer from another hospital, tracheal intubation for more than 24 hours, and prolonged stay were independently associated with hospital infection.^22^Older individuals often require hospitalization to manage their medical conditions; however, infections acquired in a hospital environment are especially important in this group due to their high mortality rate.^21^

In this context, the advanced age of the current sample also influenced the patterns of deaths related to the circulatory system. In the groups organized by specific conditions, it is worth noting that HF (I50), which had the highest proportion in a Brazilian study, with a frequency of 23.26%,^5^was not the most common entry in the DCs in this sample. At the hospital level, the highest proportion of HF (I50) was observed in the HFmrEF group compared with the HFpEF group. There was also a gradient among entries of HF (I50), infectious causes, and acute and chronic ischemic diseases, with a change in frequency related to HFrEF, HFmrEF, and HFpEF, respectively. The presence of these causes of death also confirms that the risk of death after hospitalization due to HF remains high 12–18 months after the event.^23^Rates of HF readmission in young adults are similar to those in older individuals, suggesting that the risk of rehospitalization is present regardless of age.^24^

Advanced age has also been recognized as more frequent in HFpEF compared with HFrEF and has been repeatedly confirmed in epidemiological studies of HFpEF.^16^

Since the 20th century, there has been a notable increase in longevity worldwide, primarily due to improved control of communicable diseases and advancements in sanitary measures, which have led to a higher prevalence of chronic diseases. However, this increase in longevity is reduced by population aging and growth, resulting in real rises in mortality from age-related cardiovascular diseases, increased tobacco use, and an atherogenic diet. However, there has been a decrease in deaths caused by cardiovascular diseases worldwide in recent years, thanks to the development of targeted treatments, which further increases life expectancy.^25 , 26^Notably, HF is a common condition with rising incidence, driven in part by an aging population. Epidemiological data confirm this trend and identify age over 65 years as a risk factor for developing HF.^27 - 33^

A previous study analyzing the same sample found no significant difference in mortality among the EF groups during a follow-up of 2.94 ± 2.55 years. The survival curve showed no difference between HFpEF and HFmrEF, nor between HFpEF and HFrEF, although a difference was observed between patients with HF with intermediate EF and HFrEF ( Figure 4 ). The study's hypothesis that EF categorization could be a predictive variable for in-hospital and late death was not supported by an analysis using machine learning. The multiple causes of death in this population were not analyzed in the study and were included in another study.^33^

The main limitations of the present study relate to the challenge of completing DCs, which affects the quality of information on causes of death. Another relevant limitation is that data from 21 DCs in the sample were missing, preventing a complete interpretation. However, data were obtained from 266 DCs (93%), totaling 977 entries, and there was no impact on their clinical interpretation.

This study contributes to a broader understanding of the various factors associated with HF, in particular its relationship with EF and age-related chronic diseases. To date, no studies have linked mortality from multiple causes to EF. This analysis has improved insight into the mechanisms behind HF and chronic diseases and may lead to hypotheses for developing treatment strategies to prevent morbidity and mortality.

## Conclusion

This study highlights the importance of analyzing multiple causes of death, which can uncover important aspects of the clinical course and prognosis of patients with HF that are not captured when only the underlying causes of death are analyzed. In this sample, a high occurrence of multiple causes of in-hospital and late deaths unrelated to the circulatory system was observed in patients admitted for decompensated HF, regardless of whether they had reduced EF (HFrEF), mildly reduced EF (HFmrEF), or preserved EF (HFpEF). These causes seem to be primarily linked to advanced age and chronic comorbidities rather than the HF syndrome itself. Prospective studies are necessary to confirm these findings and to determine the most suitable strategies for each HF phenotype.

Data Availability

The underlying content of the research text is contained within the manuscript.

## *Supplemental Materials
